# Skeletal muscle metastases in neuroblastoma share common progenitors with primary tumor and biologically resemble stage MS disease

**DOI:** 10.3389/fonc.2022.1106597

**Published:** 2023-01-05

**Authors:** Christina Fong, Brian H. Kushner, Angela Di Giannatale, Gunes Gundem, Shanita Li, Stephen S. Roberts, Ellen M. Basu, Anita Price, Nai-Kong V. Cheung, Shakeel Modak

**Affiliations:** ^1^ Department of Pediatrics, Memorial Sloan Kettering Cancer Center, New York, NY, United States; ^2^ Children’s Cancer and Blood Foundation Laboratories, Departments of Pediatrics, and Cell and Developmental Biology, Weill Cornell Medical College, New York, NY, United States; ^3^ Department of Epidemiology and Biostatistics, Memorial Sloan Kettering Cancer Center, New York, NY, United States; ^4^ Department of Radiology, Memorial Sloan Kettering Cancer Center, New York, NY, United States

**Keywords:** neuroblastoma, metastasis, skeletal muscle metastasis, pediatric oncology, chemotherapy, tumor evolution

## Abstract

**Introduction:**

While subcutaneous metastases are often observed with stage MS neuroblastoma, an entity that usually resolves spontaneously, skeletal muscle metastases (SMM) have been rarely described. The purpose of this retrospective study was to investigate the significance of SMM in neuroblastoma.

**Patients and methods:**

Seventeen patients with neuroblastoma SMM were diagnosed at a median age of 4.3 (0.1-15.6) months. All had SMM at diagnosis and metastases at other sites. Fifteen (88%) had ≥ 2 SMM in disparate muscle groups. One, 14, and 2 patients had low, intermediate, and high-risk disease respectively. Fifteen tumors had favorable histology without MYCN amplification, and 2 were MYCN-amplified. Most SMM (80%; n=12/15 evaluated) were MIBG-avid.

**Results:**

Only 1 patient (with MYCN-non-amplified neuroblastoma) had disease progression. All survive at median follow-up of 47.9 (16.9-318.9) months post-diagnosis. Biological markers (histology, chromosomal and genetic aberrations) were not prognostic. Whole genome sequencing of 3 matched primary and SMM lesions suggested that both primary and metastatic tumors arose from the same progenitor. SMM completely resolved in 10 patients by 12 months post-diagnosis. Of 4 patients managed with watchful observation alone without any cytotoxic therapy, 3 maintain complete remission with SMM resolving by 5, 13, and 21 months post-diagnosis respectively.

**Conclusions:**

Children with neuroblastoma SMM have an excellent prognosis, with a clinical course suggestive of stage MS disease. Based on these results, the initial management of infants with non-MYCN-amplified NB with SMM could be watchful observation, which could eliminate or reduce exposure to genotoxic therapy.

## Introduction

Neuroblastoma, the most common extracranial solid tumor of childhood, typically develops along the path of migration of neural crest cells, usually in the abdomen or mediastinum (1). Common sites of metastases include bone, bone marrow (BM), lymph nodes, and liver. Rarely, neuroblastoma metastasizes to pleura, lung, central nervous system (CNS), or testis. Although preclinical studies indicate that neural crest cells can interact with skeletal muscle progenitors and influence myogenesis *via* BMP, WNT, and NOTCH signaling pathways (2), skeletal muscle metastases (SMM) are extraordinarily rare. Only a single case of SMM in a patient with neuroblastoma has been reported: an infant with skeletal and cardiac muscle involvement treated with intermediate-dose chemotherapy who maintained remission >12 months from diagnosis (3). Two additional cases of primary muscle neuroblastoma without metastases have been described (4, 5). Among adult cancers, SMMs are rare, partly because of mechanical stress, increased lactic acid and decreased pH (6). When present in adults, they are usually symptomatic of grave prognosis, and treatment is primarily palliative (7).

While metastatic neuroblastoma has been a therapeutic challenge, a unique group of patients diagnosed in infancy with metastases to liver, skin, and BM have a favorable prognosis, often with spontaneous resolution of all sites of disease. Initially described in 1971, this special, Evans stage IV-S of neuroblastoma was defined by the presence of a small adrenal primary tumor and metastases to the liver, skin and BM without cortical bone involvement and with limited BM involvement among infants <12 months of age (8). However, computed tomography (CT), magnetic resonance imaging (MRI), and scintigraphy with meta-iodobenzylguanidine (MIBG) and FDG positron emission tomography (PET) were unavailable for routine staging in the 1970s with unusual sites of metastases likely going undetected. IV-S neuroblastoma was later incorporated into the International Neuroblastoma Staging System (termed stage 4S) and in 2009 into the International Neuroblastoma Risk Group (INRG) risk-stratification system and renamed stage MS, without materially changing its definition, except that diagnosis be made at <18 months of age (9, 10). SMM were not included in stages IV-S, 4S or MS definitions. Therefore, the clinical significance of SMM and their impact on risk assessment is unclear.

Several studies have recently highlighted the importance of better understanding spatial and temporal heterogeneity of primary tumor and distant metastasis, specifically pertaining to segmental chromosomal aberrations, *MYCN*, and various mutations including *ALK* (11, 12). The current literature comparing matched primary-relapse samples using whole exome or whole genome sequencing (WGS) suggests that branched evolution and clonal evolution of specific mutations leads to the clonal heterogeneity seen in relapsed neuroblastoma (13, 14). This clonal relationship between the primary tumor and SMM and other metastases noted in stage MS disease is unclear. We reviewed cases seen at our center and analyzed paired diagnostic and SMM samples in order to develop a better understanding of outcomes and clonality for this rare entity.

## Materials and methods

### Patients

Inclusion criteria for this retrospective review included patients diagnosed with neuroblastoma and SMM treated between 1995 and 2021 at our institution. Clinical and biological data were analyzed. Staging was recorded using the INRG staging system and response using International Neuroblastoma Response Criteria (10, 15). Progression-free survival (PFS) was defined as time from diagnosis to relapse or progression. Overall survival (OS) time was censored at date of last contact. Survival was analyzed by the Kaplan-Meier method. Approval from the Institutional Review Board of Memorial Sloan Kettering Cancer Center (MSKCC) was obtained prior to initiating this retrospective review of patient data.

### Tumor sequencing

Targeted tumor sequencing was performed using previously described methods, i.e., MSK-IMPACT (16) or Foundation One platforms (17). For WGS, tumor DNA was extracted from fresh frozen or OCT-embedded tissue biopsies and matched normal DNA from buffy coat using the DNeasy Blood & Tissue Kit (Qaigen). FFPE tissues were deparaffinized and DNA eluted as previously described (17). Briefly, genomic DNA was sheared, and sequencing libraries were prepared using the KAPA Hyper Prep Kit (Kapa Biosystems KK8504) with modifications. Libraries were subjected to a 0.5X size selection using AMPure XP beads (Beckman Coulter) after post-ligation cleanup. Libraries were not amplified by PCR and were pooled equivolume for sequencing. Samples were run on a NovaSeq 6000 (Illumina). Tumors were covered to an average of 80-90X, and normals covered at 42-52X.

### Bioinformatic analysis

Analysis of WGS data was executed using Isabl platform (18). Briefly, upon completion of each sequencing run, Isabl imports paired tumor-normal FASTQ files, executes alignment and quality control algorithms, extracts signatures (i.e., mutation signatures, microsatellite instability score, gene expression), and generates tumor purity and ploidy estimates. For samples with sufficient coverage (>60x) and tumor purity (>20%) Ensembl Variant calling is performed. High confidence somatic mutations were classified with regards to their putative role in cancer pathogenesis and microsatellite instability scores, and mutation signatures were statistically derived. Clinical relevance of mutations in common cancer genes was annotated using OncoKb, COSMIC, Ensembl Variant Effect Predictor, VAGrENT, gnomAD and ClinVar databases. Signals across data modalities (germline, somatic mutations, somatic signatures, copy number segments and gene expression profiles) were integrated for analysis.

### Inference of clonal structure

Clonal structure was analyzed using the union of high confidence SNVs from all biopsies for a patient. DPClust (v0.2.2, https://github.com/Wedge-Oxford/dpclust) was used for calculation of cancer cell fraction corrected for purity and local copy number as well as clustering and assignment of mutations across samples with the exception of the Gibbs Sampling Dirichlet Process step which has been rewritten internally for performance (19).

## Results

### Clinical and biological features

Seventeen patients (10 male; 7 female) with SMM detected at diagnosis were identified. Median age was 4.3 (range 0.1-15.6) months. Clinical and biologic features, involved muscles, and therapies administered are described in **Table 1**. Most patients presented with abdominal distension or cutaneous nodules. One patient each also had poor feeding, orbital swelling and widespread bruising. In general, disease-related symptoms at diagnosis were not severe, and no patient required ventilator support. All had stage M neuroblastoma, stratified as high (n=2), intermediate (n=14), or low-risk (n=1) (20) Most patients (15/17; 88%) had multiple SMM (median 3, range 1-8) in widely disparate muscle groups detected by CT or MRI. Frequently involved muscle groups included the gluteus, paraspinal, psoas, abdominal wall, quadriceps, hamstring, adductor, and abductor muscles. The maximum diameter ranged from 0.8-5.3 (median 2) cm. The majority (80%; n=12/15 tested) of SMM were MIBG-avid. The three MIBG-non avid SMM had maximum diameters of >1cm, and their corresponding primary tumors were MIBG-avid. Two patients did not undergo MIBG scans at diagnosis. Additional metastases were noted in bone (n=12), lymph node (n=10), liver (n=11), skin (n=11), BM (n=4), lung/pleural (n=4), dura (n=4), spleen (n=1), and CNS parenchyma (n=1).

**Table 1:** Clinical Presentation, Diagnostic Imaging, Pathology, and Outcomes of Patients with SMM.

No	Clinical Presentation				Imaging		Biology					Treatment and outcome			
	Age at Dx (mo)	Presenting symptom	Other sites of metastases	Muscles involved	Max D (cm)	MIBG	Muscle Histology	Shimada	Ploidy	Cytogenetics	Targeted Gene Sequencing	Treatment**	PFS	OS	SMM status
1	0.1	bladder mass on prenatal US	bone, bone marrow, liver, skin	gluteal, paraspinal	0.8	pos	n.d.	U	n.d.	MYCNA	negative	HD	20.7+	20.7+	CR
2	1.2	hip/ear mass	bone, dura, liver, lymph node, pleura, skin	gluteus medius, psoas, paraspinal, oblique, temporal, trapezius	5.3	pos	GN	F	1	1p loss	Somatic ALK F1245I Mutation	int-risk +2nd line	2.8	89.5+	CR
3	2.2	skin nodules	bone, dura, lymph node, skin	temporalis	1.8	pos	NBL	F	1.38	None	n.d.	int-risk	217.7+	217.7+	CR
4	2.3	poor feeding	liver,lymph node, spleen	paraspinal, gluteus, quadriceps, rectus femoris, semitendinosus	4.2	pos	n.d.	F	1	None	n.d.	None	137.5+	137.5+	PR
5	2.5	skin nodules	skin	paraspinal, chest and abdominal wall musculature	2.8	neg	NBL	F	1	None	n.d.	int-risk	119.1+	119.1+	CR
6	3.3	fever	lymph node, pleura	abdominal wall, psoas, paraspinal	3.4	pos	GN	F	1	1p, 11q loss	Somatic ALK F1174V mutation	int-risk +2nd line	110.9+	110.9+	PR
7	3.4	abnormal bruising	bone, bone marrow, dura, liver, skin	abdominal wall, gluteus maximus, labium majus, vastus lateralus	1	neg	n.d.	F	n.d.	MYCNA	n.d.	HD	15.9+	15.9+	PR
8	3.5	fever, eye swelling, hepatomegaly	bone, bone marrow, liver	gluteus medius, gluteus maximus, paraspinal	4.5	neg	NBL	F	1	None	negative, germline not performed	int-risk +2nd line	68.0+	68.0+	PR
9	4.3	skin nodules	bone, liver, lung, skin	paraspinal	2	PET pos	n.d.	F	n.d.	none	Somatic ALK 1275Q Mutation	int-risk	11.0+	11.0+	CR
10	4.5	skin nodules	liver, lymph node, skin, pleura	masseter, paraspinal	2	n.d.	n.d.	F	1.3	none	n.d.	None	305.5+	305.5+	CR
11	4.8	groin mass	bone,dura, liver, lymph node, skin	abdominal wall, adductor,gluteus, hamstring, masseter quadricep,paraspinal, psoas	1.9	pos	NBL	F	n.d.	1p loss	negative	int-risk +2nd line	21.2+	21.2+	CR
12	7.7	abdominal mass	skin	abductor magnus, gluteus, pterygoid, pectoralis, temporalis, vastus lateralis	2.2	pos	NBL	F	1.5	None	negative	None	30.8+	30.8+	CR
13	9.5	abdominal mass	bone, liver, lymph node, skin	paravertebral, gluteus	1.6	pos	n.d.	F	n.d.	11q, 17q gain	negative	int-risk	34.7+	34.7+	PR
14	10.6	abdominal mass	bone, bone marrow, brain, lung, liver, lymph node, skin	gluteus, thoracic wall, proximal thigh	1.8	pos	NBL	F	n.d.	1p loss	Germline RECQL4 mutation	int-risk +2nd line	17.1+	17.1+	CR
15	10.8	abdominal mass	bone, lymph node	soleus, vastus lateralis, gluteus medius, biceps femoris, adductor, semimembranosis	3	pos	n.d.	F	n.d.	17q gain	Somatic KNSTRN C62F mutation	None	27.6+	27.6+	CR
16	15.6	fever, back mass	bone, liver, lymph node	paraspinal, spinous rectus, trapezius,	1.9	pos	NBL	F	1.4	11q loss	Somatic missense mutation SMRACa4a, ZFHx3. Germline CHEK2 mutation	int-risk	15.2+	15.2+	PR
17	8.9	abdominal mass	Bone, liver, lymph node, skin	Paraspinal, semitendinosus	1.7	Pos	GN	F	1.93	None	n.d	int-risk	12.3+	104.4+	CR

Patient No (No); Age at Diagnosis (Dx); Months (mo); Skeletal Muscle Metastasis (SMM); Maximum SMM Diameter (Max D); Positive (Pos); Neuroblastoma (NBL); Ganglioneuroma (GN); Intermediate Dose (int-risk); Second Line (2nd line); High Dose (HD); Not Done (n.d.); Unfavorable (U); Favorable (F); Complete Remission (CR); Partial Remission (PR); **No patient received radiotherapy.

Ten patients underwent biopsies of SMM. Seven biopsies directly correlated with the histopathology of the primary tumor, i.e., favorable histology neuroblastoma. Three biopsies (from patients #2, #6, and #17) revealed ganglioneuroma without immature elements while the matched primary tumor showed partially differentiated neuroblastoma. Fifteen (88%) primary tumors had favorable histology without *MYCN*-amplification. Two tumors were *MYCN*-amplified. Half (5/10 tested) of the tumors were hyperdiploid, and 41% (7/17) had aberrations of chromosome 1p, 11q and/or 17q. Targeted exome sequencing on primary tumors detected somatic *ALK* mutations in 3/10 tested (F1174V, F1245I, 1275Q). One patient each had germline *RECQL4* and *CHEK2* mutation.

### WGS

**Figure 1:**
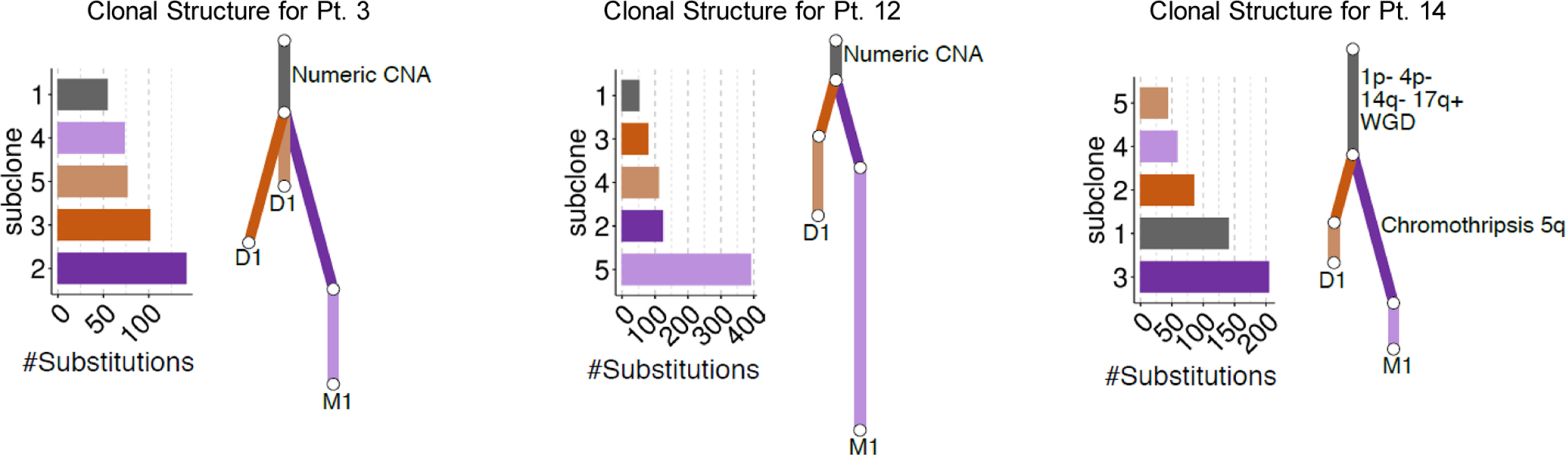
Summary of the clonal relationship between the diagnostic (D1) and muscle metastatic (M1) tumors for each patient. Mutation clusters identified in each patient are shown bar plots. Inferred clonal relationship between clusters is summarized in the form of a phylogenetic tree where branch length is proportional to the number of mutations in the cluster. Mutations shared fully clonally in both tumors are shown in dark grey followed by tumor-specific subclones.

Three patients (**Table 1**, patients #3, #12, #14) had both a primary diagnostic sample and muscle metastasis sample available for WGS analysis. By comparing the matched tumors in each patient, we identified a set of substitutions found on the trunk that were fully clonal in both the primary and metastatic tumor (**Figure 1**). Overall, truncal substitutions as well as the shared copy number aberrations (CNAs) designate the most recent common ancestor shared between tumor cells in the primary sample and cells leading to muscle metastasis. In two patients (#3, #12) with numeric CNAs only, the genome-wide CNA profile was the same in both primary and metastatic tumor. In another patient (#14), we observed segmental CNAs including losses on 1p and 4p as well as potential whole genome duplication. Of note, the clone leading to the muscle metastasis had a complex structural rearrangement in the form of a chromothripsis on chromosome 5q. SMM resolved in all three patients, one of whom was managed with observation only without cytotoxic therapy (patient #12).

### Management and outcome

At diagnosis, patients with *MYCN*-non-amplified (*MYCN*-NA) neuroblastoma received no cytotoxic chemotherapy (n=4) or intermediate-risk chemotherapy (n=11) (**Table 1**). Whereas the standard initial approach at MSKCC for patients without severe symptoms would be watchful observation without cytotoxic therapy, most (n=11) patients were referred after starting treatment at other centers where initial treatment decisions were made based on local protocols. One patient (#2), who was initially managed with observation alone, relapsed 2.4 months post-diagnosis (but not in muscle), subsequently received intermediate-dose chemotherapy and has maintained remission 87+ months later. Of the 15 patients with MYCN-NA neuroblastoma, 10 and 5 demonstrated complete and partial resolution of SMM respectively. No patient received dose-intensive chemotherapy, radiotherapy or immunotherapy.

Both patients with *MYCN*-amplified disease had multiple sites of metastases in addition to SMM (Patients #1 and #7). Both achieved remission with high-dose induction chemotherapy and surgery. One (#1) was consolidated with naxitamab-based immunotherapy (21) and a bivalent gangliosides vaccine (22), without autologous transplant or radiotherapy, and the other (#7) with myeloablative chemotherapy, radiotherapy and dinutuximab-based immunotherapy. Both remain in remission 27+ and 15+ months post-diagnosis.

**Figure 2:**
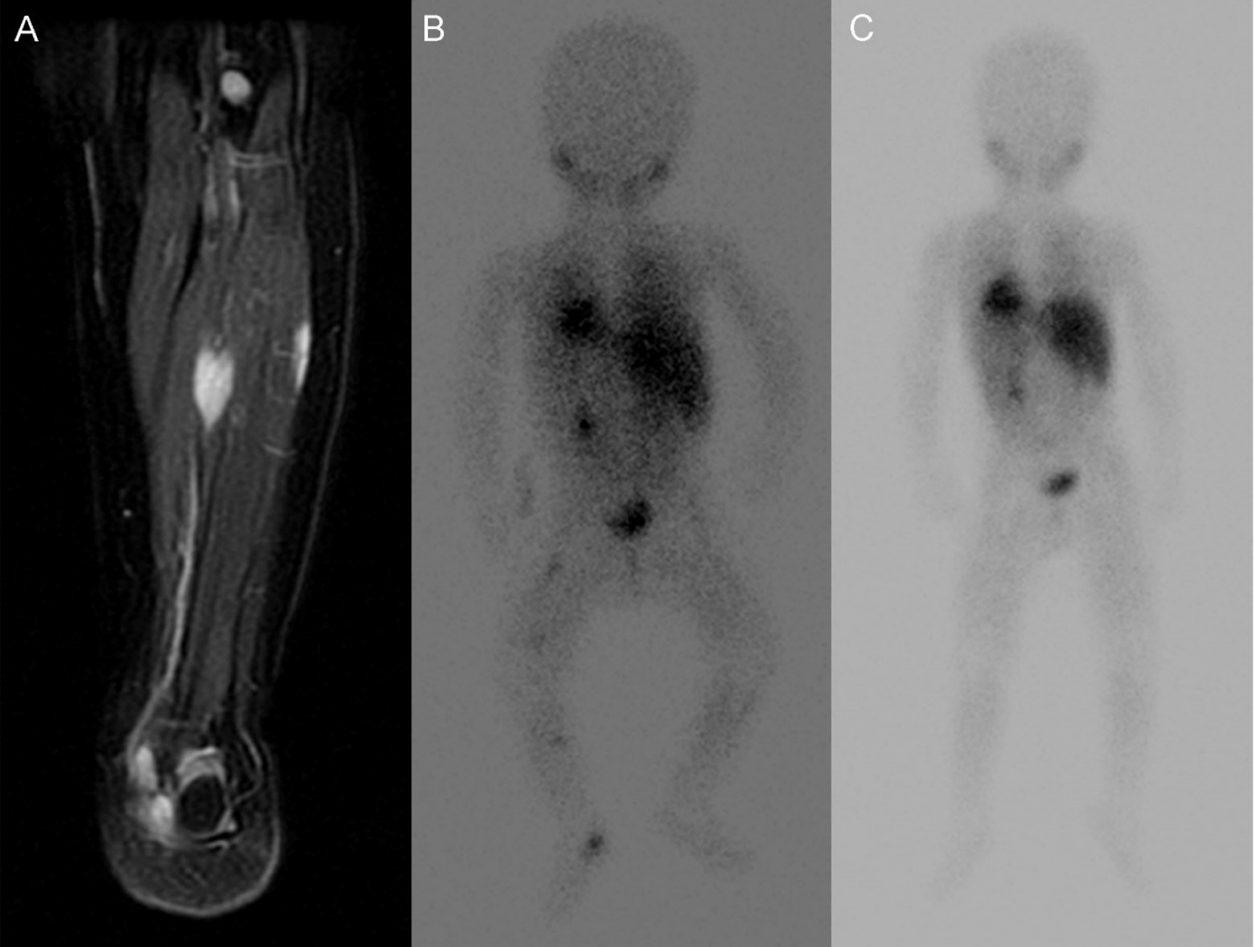
Images of lower extremity SMM of Patient No. 15. **(A)** T2 MR of SMM at diagnosis **(B)** corresponding MIBG avid SMM at diagnosis. **(C)** MIBG show resolution of SMM without treatment after 21 months.

We noted 3-year PFS and OS of 87% and 100% respectively. Median PFS for the entire group is 40.1 (12.3-318.2) months. All patients survive at a median follow up of 47.9 (16.9-318.9) months after diagnosis. Eleven patients are in complete remission including complete resolution of SMM at 12 months post-diagnosis (three patients managed with observation alone, #10, #12, and #15) (**Figure 2** [patient #9]). Median time to resolution of SMM was 10.5 (range 5 – 21) months. The remaining 6 patients have completed therapy but have persistent, non-progressive disease with decreasing size of SMM. Of the 4 patients managed with watchful observation alone without any cytotoxic therapy, one is in partial remission and 3 maintain complete remission with skeletal muscle metastases resolving by 5, 13, and 21 months post-diagnosis respectively.

## Discussion

The understanding of SMM in neuroblastoma remains limited due to their rarity. Our data substantially improves this understanding by identifying skeletal muscle as unique sites of metastasis. SMM share a close clonal relationship to primary tumors and are associated with an excellent prognosis. SMM were diagnosed at <18 months in all patients analyzed and were accompanied by metastases at sites typical for high-risk neuroblastoma (bone or BM) or at atypical sites (dura, pleura, or CNS). Therefore, our patients did not meet the criteria for classical stage MS disease. Nevertheless, the clinical course of our patients resembled that of stage MS disease with an excellent survival outcome despite receiving no chemotherapy or truncated intermediate-risk chemotherapy regimens (for those with *MYCN*-NA disease) (23). Furthermore, biological markers including chromosomal aberrations, ploidy, or *ALK* mutations did not impact outcome. The 2 patients with *MYCN*-A disease remain progression-free, one despite not having undergone myeloablative transplant or radiotherapy (patient #1). Of the 4 patients managed without any cytotoxic therapy, 3 achieved and maintain remission in all disease sites and the fourth, treated with intermediate-dose chemotherapy after relapse, has not had further disease progression for 87+ months. Of note, among the 4 patients not treated at diagnosis, the SMMs continued to regress, similar to regressions of disease seen in patients with classical MS disease.

The young age at diagnosis of these synchronous lesions could lead to speculation that they represent multiclonal disease as has been suggested for synchronous bilateral neuroblastoma (24). These were considered to be independent neoplasms that separated early in embryogenesis and developed in parallel. In contrast, using whole exome and whole genome sequencing, several studies have identified common mutations and rearrangements between primary and metastatic biopsy samples in high-risk stage M neuroblastoma suggesting that the two are clonally related (11, 13, 14). The limited availability of both diagnostic and metastatic disease specimens for patients with stage MS disease has hindered the investigation of clonality in patients with stage MS disease. Our genomic analysis of paired primary tumor biopsies and SMM biopsies demonstrated a close clonal relationship between the primary tumor and the SMM. Based on identification of shared truncal substitutions and CNAs in primary and SMM biopsies, both the primary tumor and SMM appeared to have arisen from a common tumor cell progenitor – from a monoclonal cell of origin. Interestingly, 2 patients had SMM with ganglioneuroma without immature elements despite their matched primary tumor biopsy showing partially differentiated neuroblastoma. These patients each had involvement of >3 muscle groups, 1p chromosomal aberrations and *ALK* mutations and are alive progression free at 89+ and 110+ months post-diagnosis with remission of SMM. The discrepancy in histological findings between primary and metastatic tumor could be attributed to different rates of tumor-cell maturation in different sites. Further genomic, epigenomic, transcriptomic or proteomic studies comparing primary versus SMM lesions in a larger sample size might shed light on a signature for diagnosing MS disease, possibly uncovering candidate genes or pathways to study spontaneous regression of human cancer.

We identified a subgroup of patients all diagnosed at a favorable age with a rare clinical presentation of SMM who did not meet criteria for stratification as stage MS. However, we observed a favorable outcome both for survival and resolution of disease that more closely parallel that of stage MS disease, even when no cytotoxic therapy was administered. This was a retrospective study of patients treated at a single tertiary center and our cohort included several patients who received initial therapy at other institutions. Although a larger cohort would help confirm our findings, SMM in neuroblastoma are extraordinarily rare events. The excellent outcome in all patients, and the spontaneous regression of many SMM and other metastases in some of our patients suggest that SMM constitute a *forme fruste* of classical stage MS disease associated with spontaneous resolution. Reducing cytotoxic therapy without compromising cure is an unmet need in treating metastatic pediatric cancers. At our institution we have a long-standing practice of avoiding cytotoxic therapy for non-high-risk neuroblastoma. While withholding all chemotherapy may not be feasible for all patients with SMM, the results presented here suggest that the presence of metastases in skeletal muscles should not lead to upstaging patients, rather it should strengthen the MS staging where a wait-and-watch approach could be justified.

## Data availability statement

The original contributions presented in the study are included in the article/supplementary materials. Further inquiries can be directed to the corresponding author.

## Ethics statement

The retrospective analysis of patient data was approved by the Institutional Review Board of Memorial Sloan Kettering Cancer Center (MSKCC). Written informed consent was not required.

## Author contributions

CF drafted the original manuscript. BK, AD, SM contributed to the conception of the work and the acquisition of data. All authors provided critical review and revisions for important intellectual content; the work reported in the paper has been performed by the authors, unless clearly specified in the text. All authors contributed to the article and approved the submitted version.
